# Exploring the links between various traumatic experiences and ICD-11 PTSD and Complex PTSD: A cross-sectional study

**DOI:** 10.3389/fpsyg.2022.896981

**Published:** 2022-09-15

**Authors:** Agniete Kairyte, Monika Kvedaraite, Evaldas Kazlauskas, Odeta Gelezelyte

**Affiliations:** Center for Psychotraumatology, Institute of Psychology, Vilnius University, Vilnius, Lithuania

**Keywords:** trauma exposure, trauma type, posttraumatic stress, complex posttraumatic stress, ICD-11, risk factors

## Abstract

**Background:**

The 11th revision of the International Classification of Diseases (ICD-11) included two distinct trauma-related diagnoses—Posttraumatic Stress Disorder (PTSD) and Complex Posttraumatic Stress Disorder (CPTSD). The initial diagnostic factor for both disorders is exposure to a traumatic event. This study aimed to explore whether exposure to different traumatic experiences distinguish risk for PTSD and CPTSD.

**Methods:**

The study sample comprised 158 trauma-exposed participants, *M*(SD)_age_ = 33.61(9.73). The Life Events Checklist-Revised (LEC-R) was used to evaluate trauma exposure, and the International Trauma Questionnaire (ITQ) was used to assess risk for ICD-11 PTSD and CPTSD. Multinomial logistic regression was used to determine traumatic events as predictors of risk for PTSD and CPTSD.

**Results:**

Analysis revealed that sexual abuse experienced in childhood or adulthood was associated with both PTSD and CPTSD. History of other unwanted sexual experiences and childhood physical abuse predicted CPTSD compared to PTSD, whereas exposure to natural disasters predicted PTSD compared to CPTSD.

**Conclusions:**

The results showed that experiences of certain traumatic events, such as sexual trauma, childhood physical abuse or natural disasters, might help distinguish risk for PTSD and CPTSD. Nevertheless, future studies on specific aspects of trauma exposure are necessary.

## Introduction

Traumatic experiences are defined as an event or series of events of an extremely threatening or horrific nature (Brewin et al., 2017). Those events may include physical assault, sexual abuse, transportation accident, natural disaster, sudden, unexpected loss of a loved one, captivity, and other health- or life-threatening events. Traumatic experiences are highly prevalent. Various studies showed that about 70% of the adult population had experienced at least one traumatic event (Benjet et al., 2016; Kessler et al., 2017), with an average of 3.2 different trauma exposures during the lifetime (Kessler et al., 2017). The most prevalent traumatic experiences vary through different studies and cultures. Nevertheless, the most prevalent experiences often reported in different studies are sudden, unexpected loss of a loved one, transportation accident, severe human suffering, physical assault, and childhood physical abuse (Kazlauskas et al., 2018; Cloitre et al., 2019; Kvedaraite et al., 2021). Such life-threatening experiences may lead to various levels of psychological reactions and disturbances, such as trauma-related disorders.

The 11^th^ revision of the International Classification of Diseases (ICD-11; World Health Organization, 2018) proposed two distinct diagnoses which are directly linked to trauma exposure—posttraumatic stress disorder (PTSD) and complex posttraumatic stress disorder (CPTSD). Both these trauma-related disorders are caused by traumatic experiences, but they are symptomatically distinct. PTSD includes symptoms of (1) re-experiencing the traumatic event or events in the present in the form of vivid intrusive memories, flashbacks, or nightmares, (2) avoidance of the reminders of the traumatic experience, and (3) persistent perception of the heightened current threat. Those symptoms have to persist for at least several weeks and cause significant impairment in important areas of functioning. CPTSD includes all symptoms of PTSD as well as symptoms of disturbances in self-organization (DSO). DSO symptoms include: (1) problems in affect regulation, (2) beliefs about oneself as diminished, defeated or worthless, and (3) difficulties in sustaining relationships and feeling close to others. In order to diagnose CPTSD, DSO symptoms have to cause significant impairment in important areas of functioning (World Health Organization, 2018). Population-based empirical research in different countries showed that the prevalence of the risk for PTSD was 3.4–5.8% and for CPTSD it was 1.8–7.0% (Cloitre et al., 2019; Hyland et al., 2021; Kvedaraite et al., 2021).

In the ICD-11, it is defined that both PTSD and CPTSD may develop following exposure to an extremely threatening or horrific event or series of events. In addition, it is also indicated that CPTSD is more related to prolonged or repetitive traumatic events from which escape is difficult or impossible (e.g., torture, slavery, genocide campaigns, prolonged domestic violence, and repeated childhood sexual or physical abuse; World Health Organization, 2018). However, these types of experiences are not a necessary diagnostic requirement for CPTSD. There is a lack of empirical evidence that prolonged or repetitive traumatic experiences are more related to CPTSD as well. The distinction of traumatic exposure which would be more characteristic to PTSD or CPTSD might facilitate the identification of these different disorders. However, until the ICD-11 is implemented, most studies have been based on relations between PTSD and different traumatic experiences (e.g., Forbes et al., 2014; Kessler et al., 2017; Oakley et al., 2021). Therefore, changes in the new revision of the ICD-11 demand studies exploring if various types of trauma exposure are related to different symptom profiles of PTSD and CPTSD.

Research conducted so far demonstrated inconsistent findings regarding the associations between exposure to different traumatic events and risk for PTSD or CPTSD. Results of previous studies showed that CPTSD might be linked with traumas of interpersonal type, which are characterized by perpetrators inflicting suffering as well as control over their victim (Palic et al., 2016), such as physical assault, childhood (Hyland et al., 2017; Cloitre et al., 2019; Gilbar et al., 2019) or adulthood sexual abuse (Redican et al., 2022). Nevertheless, in a different study, Redican et al. (2022) observed that physical abuse in childhood was related to PTSD but not CPTSD. Also, non-interpersonal experiences, which are not caused by another human being, such as near-drowning experience, were related to CPTSD in the study by Hyland et al. (2017). Moreover, some studies have also found that a higher number of different traumatic experiences predicted both PTSD and CPTSD (Cloitre et al., 2019; Karatzias et al., 2019; Kira et al., 2022; Redican et al., 2022).

These inconsistencies in previous research findings led to the current study that aimed to investigate the differences in traumatic exposure between PTSD and CPTSD risk groups in order to determine which traumatic events might distinguish risk for ICD-11 PTSD and CPTSD in the trauma-exposed sample.

## Materials and methods

### Participants and procedure

The study was approved by the Vilnius University Research Ethics Committee (16.01.2020, No. 33). Participants were invited to take part in the study *via* social media platforms and mental health care professionals in various regions of Lithuania. Inclusion criteria for the study were: (1) ≥ 18-years-old, (2) experience of at least one potentially traumatic event during lifetime. Participants were asked to fill in an online registration form to determine compliance with the inclusion criteria. Individuals included in the study were asked to fill in an online survey on a secure survey platform. All participants provided informed consent at the beginning of the survey. After filling out the survey, participants were provided with information regarding the available mental health services in Lithuania. The data were collected between October 2020 and June 2021.

Overall, 192 people registered to participate in a study. Of them, 22 (11.46%) did not meet the inclusion criteria, and 12 (6.25%) did not fill in the survey. The final self-referred trauma-exposed study sample comprised 158 adults aged from 18 to 58, *M*(SD)_age_ = 33.61(9.73). The majority of participants were female (*n* = 135; 85.4%), living in an urban area (*n* = 140; 88.6%), working and/or studying (*n* = 131; 82.9%). Almost half of the sample has been in a long-term relationship (*n* = 78; 49.4%). More than one-third have been currently seeing a mental health specialist (*n* = 58, 36.7%), and more than a third had contacted a mental health professional earlier in life (*n* = 57, 36.1%).

## Measures

### Exposure to traumatic events

The revised version of the Life Events Checklist (LEC-R; Weathers et al., 2013) was used to evaluate lifetime exposure to traumatic experiences. The checklist is comprised of 18 potentially traumatic events. Participants were asked if they had experienced any of these events, with possible answers of “Happened to me,” “Witnessed it,” “Learned about it,” “Not sure,” “Does not apply to me.” The event was coded as experienced if it happened or was witnessed by the participant. The cumulative trauma was calculated by summing all experienced different traumatic events. LEC-R was used in previous studies in Lithuania (Kazlauskas et al., 2018; Kvedaraite et al., 2021).

### Posttraumatic stress and complex posttraumatic stress

The International Trauma Questionnaire (ITQ; Cloitre et al., 2018) was used to measure the risk for ICD-11 PTSD and CPTSD. The ITQ comprises two sections for PTSD and DSO symptom evaluation within three symptom clusters per each and two items per cluster. PTSD symptom clusters include the evaluation of re-experiencing, avoidance, and sense of current threat symptoms. Items in the ITQ DSO symptom clusters evaluate symptoms of affective dysregulation, negative self-concept, and disturbances in relationships. Both sections of PTSD and DSO are followed by three functional impairment questions measuring how these symptoms had affected social, working, or other important areas of a person’s life. Participants were asked to evaluate how much they had been bothered by these symptoms in the past month on a five-point Likert scale from “Not at all” (=0) to “Extremely” (=4). The criteria for PTSD risk are considered met if at least one symptom in each cluster of PTSD section had been experienced moderately and at least one life area had been moderately affected by any of these symptoms (≥ 2). The criteria for CPTSD risk are met if PTSD criteria had been met and at least one symptom in each cluster of DSO symptom section had been evaluated as moderately true, and at least one life area had been affected by these symptoms at least moderately (≥ 2). Based on ICD-11, a person cannot be diagnosed with PTSD and CPTSD simultaneously. If the person meets the criteria for the risk for CPTSD, PTSD risk is not considered anymore. Previous studies demonstrated good psychometric properties of the Lithuanian version of the ITQ (Kvedaraite et al., 2021). The reliability of the ITQ in this study was good, Cronbach’s *α* = 0.87 (for PTSD section *α* = 0.81, DSO *α* = 0.83).

## Data analysis

The data analyses were conducted using IBM SPSS version 25.0. Descriptive statistics were used for the description of demographic information and estimating the prevalence of the traumatic events in the study sample. Chi-square tests were used to determine differences in the prevalence of trauma exposure among no PTSD/CPTSD, PTSD risk and CPTSD risk groups. Multinomial logistic regression was used to evaluate which types of trauma exposure have significant predicting value on PTSD and CPTSD risks. The multinomial logistic regression was applied twice, with firstly no PTSD/CPTSD group set as a reference group and further PTSD risk group set as a reference group.

## Results

More than one-third (*n* = 59; 37.3%) of the study sample met the criteria for CPTSD and 14.6% (*n* = 23), for PTSD risk. The mean of different traumatic experiences in the sample was 5.40 (SD = 2.73). The prevalence of trauma exposure and differences among groups of no PTSD/CPTSD, PTSD and CPTSD risk are presented in Table 1. The most prevalent traumatic experiences in the total sample were severe human suffering (65.8%), childhood physical abuse (62.7%), physical assault in adulthood (60.1%), other unwanted or uncomfortable sexual experiences (52.5%), and sudden, unexpected death of someone close (51.3%). The most prevalent traumatic experiences in PTSD group were physical assault (56.5%), severe human suffering (56.5%), childhood physical abuse (52.2%), and sudden unexpected death of someone close (52.2%). In CPTSD risk group, the most frequent traumatic events were similar: childhood physical abuse (72.9%), severe human suffering (69.5%), physical assault (67.8%), and other unwanted sexual experience (64.4%).

**Table 1:** Prevalence of traumatic events in the study sample.

	Total sample (*N* = 158)	No PTSD/CPTSD (*n* = 76)	PTSD (*n* = 23)	CPTSD (*n* = 59)	*χ*^2^(2)
	*N* (%)	*n* (%)	*n* (%)	*n* (%)	
*Traumatic events (LEC-R)*					
Natural disaster	15 (9.5)	4 (5.3)	4 (17.4)	7 (11.9)	3.64
Fire or explosion	32 (20.3)	12 (15.8)	4 (17.4)	16 (27.1)	2.78
Transportation accident	73 (46.2)	34 (44.7)	8 (34.8)	31 (52.2)	2.23
Serious accident at work, home or during recreational activity	49 (31.0)	21 (27.6)	8 (34.8)	20 (33.9)	0.79
Exposure to toxic substance	14 (8.9)	6 (7.9)	0 (0)	8 (13.6)	3.94
Childhood physical abuse	99 (62.7)	44 (57.9)	12 (52.2)	43 (72.9)	4.45
Physical assault	95 (60.1)	42 (55.3)	13 (56.5)	40 (67.8)	2.32
Assault with a weapon	28 (17.7)	12 (15.8)	3 (13.0)	13 (22.0)	1.29
Childhood sexual abuse	48 (30.4)	16 (21.1)	8 (34.8)	24 (40.7)	6.30^*^
Sexual assault	44 (27.8)	11 (14.5)	8 (34.8)	25 (42.4)	13.51^**^
Other unwanted sexual experience	83 (52.5)	37 (48.7)	8 (34.8)	38 (64.4)	6.69^*^
Combat or exposure to a war-zone	3 (1.9)	0 (0)	1 (4.3)	2 (3.4)	2.92
Captivity	6 (3.8)	2 (2.6)	3 (13.0)	1 (1.7)	6.37^*^
Life-threatening illness or injury	49 (31.0)	25 (32.9)	5 (21.7)	19 (32.2)	1.09
Severe human suffering	104 (65.8)	50 (65.8)	13 (56.5)	41 (69.5)	1.24
Sudden, violent death	24 (15.2)	13 (17.1)	3 (13.0)	8 (13.6)	0.42
Sudden, unexpected death of someone close	81 (51.3)	39 (51.3)	12 (52.2)	30 (50.8)	0.01
Serious injury, harm or death caused to someone else	6 (3.8)	3 (3.9)	0 (0)	3 (5.1)	1.18
*Cumulative trauma*					
*M* (SD)	5.40 (2.73)	4.88 (2.05)	4.91 (3.03)	6.25 (3.18)	
1	10 (6.3)	4 (5.3)	1 (4.3)	5 (8.5)	0.76
2–3	31 (19.6)	16 (21.1)	8 (34.8)	7 (11.9)	5.70
4–5	48 (30.4)	28 (36.8)	6 (26.1)	14 (23.7)	2.94
≥ 6	69 (43.7)	28 (36.8)	8 (34.8)	33 (55.9)	5.79

LEC-R, life events checklist-revised; PTSD, posttraumatic stress disorder; CPTSD, complex posttraumatic stress disorder. *p < 0.05; **p <0.01.

Significant differences of various traumatic exposure were found in the three study groups—no PTSD/CPTSD, PTSD risk and CPTSD risk. Captivity was found to be a risk for PTSD (13.0%) in comparison to CPTSD [1.7%; *χ*^2^(1) = 4.59, *p* = 0.032] and no PTSD/CPTSD groups [2.6%; *χ*^2^(1) = 3.99, *p* = 0.046]. Sexual assault was associated with PTSD risk (34.8%) in comparison to no PTSD/CPTSD risk group [14.5%; *χ*^2^(1) = 4.70, *p* = 0.030]. Furthermore, sexual trauma was associated with higher CPTSD risk. We found that childhood sexual abuse [40.7% in CPTSD, 21.1% no PTSD/CPTSD; *χ*^2^ (1) = 6.14, *p* = 0.013] and sexual assault in adulthood [42.4% in CPTSD, 14.5% in no PTSD/CPTSD; *χ*^2^ (1) = 13.22, *p* < 0.001] had significant effect on CPTSD risk. Moreover, unwanted sexual experiences were significantly more prevalent in CPTSD risk (64.4%) risk group compared to PTSD risk group (34.8%; *χ*^2^ (1) = 5.90, *p* = 0.015).

Cumulative trauma experience had a diverse effect on PTSD and CPTSD risk. Exposure to 2 or 3 traumatic experiences was associated with the higher risk for PTSD (38.8%) rather than CPTSD (11.9%; *χ*^2^ (1) = 5.82, *p* = 0.016). However, those exposed to 6 or more traumatic experiences had higher risk for CPTSD (55.9%) in contrast to no PTSD/CPTSD group (36.8%; *χ*^2^ (1) = 4.89, *p* = 0.027).

Further, we performed multinomial logistic regression to estimate the role of various traumatic experiences of PTSD and CPTSD risk. Due to the low prevalence of some traumatic experiences assessed in the sample, we excluded them from multinomial logistic regression analysis. Not included in the multinomial logistic analysis were following experiences: experiences of combat or exposure to war-zone (1.9%), captivity (3.8%), serious injury, harm, or death caused by the participant to someone else (3.8%) and exposure to toxic substances (8.9%). Thus, one participant who experienced only one of these events was excluded from the following analysis (*N* = 157).

The Multinomial logistic regression model Likelihood Ratio Tests showed good model fit [*χ*^2^(*df*) = 52.84(30), *p = 0*.006]. The *Negelkerke* determination pseudo coefficient *R*^2^ was 0.33, *Cox’s and Snell’s* 0.29. Experiences of childhood and adulthood sexual abuse were statistically significant predictors for both PTSD and CPTSD risk in comparison with no PTSD/CPTSD group. Moreover, exposure to a natural disaster was found as a significant predictor for PTSD in comparison with both no PTSD/CPTSD and CPTSD risk groups. Furthermore, exposure to serious accidents at work, home or during recreational activity significantly predicted PTSD risk vs. no PTSD/CPTSD risk. Childhood physical abuse had a significant predicting value for CPTSD risk in comparison with no PTSD/CPTSD group. Also, cumulative trauma exposure was a significant predictor for CPTSD vs. no PTSD/CPTSD risk group. Childhood physical abuse and other unwanted sexual experiences were significant predictors for CPTSD vs. PTSD risk. All results of the regression analysis are presented in Table 2.

**Table 2:** Multinomial Logistic Regression for the prediction of risk for PTSD and CPTSD (*N* = 157).

*Variable*	PTSD vs. No PTSD/CPTSD				CPTSD vs. No PTSD/CPTSD				CPTSD vs. PTSD			
	*B*	SE	*p*	OR [95% CI]	*B*	SE	*p*	OR [95% CI]	*B*	SE	*p*	OR [95% CI]
Natural disaster	3.39	1.06	0.001	29.79 [3.74–237.17]	0.65	0.90	0.472	1.91 [0.33–11.05]	−2.75	1.10	0.012	0.06 [0.01–0.55]
Fire or explosion	−0.69	0.88	0.434	0.50 [0.09–2.81]	0.84	0.57	0.142	2.31 [0.76–7.07]	1.52	0.86	0.076	4.59 [0.85–24.69]
Transportation accident	−0.82	0.75	0.275	0.44 [0.10–1.92]	0.48	0.49	0.325	1.62 [0.62–4.20]	1.30	0.76	0.086	3.68 [0.83–16.24]
Serious accident at work, home or during recreational activity	1.68	0.73	0.022	5.35 [1.28–22.39]	0.86	0.52	0.099	2.35 [0.85–6.52]	−0.82	0.73	0.260	0.44 [0.11–1.84]
Childhood physical abuse	−0.44	0.69	0.527	0.65 [0.17–2.49]	0.99	0.50	0.047	2.71 [1.02–7.26]	1.43	0.71	0.043	4.20 [1.05–16.83]
Physical assault	0.78	0.70	0.266	2.19 [0.55–8.67]	0.66	0.53	0.216	1.93 [0.68–5.49]	−0.12	0.72	0.865	0.89 [0.22–3.60]
Assault with a weapon	−1.05	0.91	0.246	0.35 [0.06–2.07]	0.18	0.58	0.752	1.20 [0.38–3.77]	1.24	0.89	0.163	3.45 [0.61–19.60]
Childhood sexual abuse	1.59	0.69	0.021	4.91 [1.27–19.05]	1.11	0.48	0.021	3.05 [1.18–7.85]	−0.48	0.66	0.470	0.62 [0.17–2.27]
Sexual assault	2.51	0.79	0.002	12.28 [2.57–58.71]	1.91	0.55	<0.001	6.73 [2.31–19.62]	−0.60	0.76	0.429	0.55 [0.12–2.43]
Other unwanted or uncomfortable sexual experience	−1.19	0.70	0.089	0.30 [0.08–1.20]	0.77	0.47	0.100	2.17 [0.86–5.44]	1.96	0.70	0.005	7.12 [1.81–27.94]
Life-threatening illness or injury	−0.41	0.69	0.555	0.67 [0.17–2.56]	0.41	0.49	0.397	1.51 [0.58–3.89]	0.82	0.68	0.229	2.26 [0.60–8.55]
Severe human suffering	−0.03	0.66	0.966	0.97 [0.27–3.52]	0.36	0.48	0.459	1.43 [0.56–3.69]	0.39	0.67	0.567	1.47 [0.39–5.52]
Sudden, violent death	−0.10	0.81	0.904	0.91 [0.18–4.48]	−0.05	0.60	0.940	0.96 [0.29–3.12]	0.05	0.85	0.950	1.06 [0.20–5.52]
Sudden, unexpected death of someone close	0.34	0.65	0.604	1.40 [0.39–5.00]	0.35	0.47	0.450	1.42 [0.57–3.54]	0.01	0.66	0.982	1.01 [0.28–3.66]
Cumulative trauma	−0.97	0.69	0.163	0.38 [0.10–1.48]	−1.49	0.54	0.006	0.23 [0.08–0.65]	−0.52	0.66	0.429	0.59 [0.16–2.17]

PTSD, posttraumatic stress disorder; CPTSD, complex posttraumatic stress disorder; OR, odds ratio; CI, confidence interval; SE, standard error.

## Discussion

This study contributes to the previous studies by providing new evidence-based knowledge about the links between exposure to various traumatic experiences and risk for posttraumatic stress disorder (PTSD) and complex posttraumatic stress disorder (CPTSD). In the current study, we found that experiences of natural disasters, serious accidents at home, work or during recreational activity, childhood physical abuse, other unwanted sexual experiences and poly-traumatization distinguished the risk for PTSD and CPTSD. The experiences of a natural disaster and serious accidents experienced at work, home or during recreational activity were associated with the elevated risk for PTSD. However, childhood physical abuse, other unwanted sexual experience and poly-traumatization were related to the increased CPTSD risk. Whereas experiences of sexual abuse in childhood or adulthood were linked with the elevated risk for both PTSD and CPTSD.

The results of our study confirm previous findings that CPTSD is related to experiences of physical abuse in childhood and higher cumulative lifetime trauma exposure (Hyland et al., 2017; Cloitre et al., 2019; Gilbar et al., 2019). Adverse experiences such as childhood physical abuse or poly-traumatization might be the core risk factors for the developmental disruptions at a young age that may lead not only to PTSD symptoms but also to disturbances in self-organization that are specific to CPTSD (Hyland et al., 2017; Kira et al., 2022). Also, the results of the current study are in line with the findings of previous research that both PTSD and CPTSD might be associated with experienced sexual abuse in childhood or adulthood (Redican et al., 2022).

Moreover, the current study results expand the knowledge that there might be probable patterns of PTSD and CPTSD development after exposure to traumatic events of non-interpersonal and interpersonal nature. In this study, we found that non-interpersonal traumas such as natural disasters or serious accidents might be more related to PTSD than CPTSD risk. In contrast, traumatic exposure of interpersonal trauma, such as physical abuse in childhood and other unwanted sexual experiences, might be more related to CPTSD risk. However, the pattern that interpersonal and non-interpersonal traumatic events would differentiate PTSD and CPTSD was not that explicit in the current study. Prior studies also demonstrated that not only specific trauma exposure by itself is a risk factor that distinguishes PTSD and CPTSD. For example, Simon et al. (2019) found that trauma-related social disapproval is associated with the higher development of CPTSD in comparison to PTSD symptoms. In a study by Kvedaraite et al. (2021), it was also demonstrated that social disapproval mediated only the relationship between trauma exposure and CPTSD but not the association between trauma exposure and PTSD. Hence, for future studies, it is necessary to analyze other potential risk and resilience factors in the development of PTSD and CPTSD symptoms, such as perceived social support, trauma disclosure, experienced stressors, and others.

A finding that other unwanted sexual experiences but not an experience of sexual assault differentiate PTSD and CPTSD had been unexpected. A possible explanation would be that for the participants it might not be easy to differentiate between sexual assault and other unwanted sexual experiences. Also, unwanted sexual experiences might be interpreted as repetitive exposure to sexual abuse for those participants who had experienced sexual abuse. In addition, experiencing unwanted sexual experiences might also be related to the DSO symptoms of CPTSD. Emotional dysregulation, negative self-concept and disturbed relationships might make a person more vulnerable and less able to protect oneself from additional traumatization by unwanted sexual experiences.

In comparison to previous research, the prevalence of PTSD and CPTSD risk in the current study sample was relatively high—14.6 and 37.6%, respectively. Such results may be firstly explained by a specific study sample of people with a history of traumatic experiences. Also, people who experience trauma-related difficulties might have been more interested in participating in the study as the declared aim of the study stated that participation might help gaining more knowledge about peoples’ posttraumatic reactions and, as a result, improve the establishment of psychological intervention methods for trauma-affected people.

According to the findings of this study, some potential clinical implications may arise. The results indicate that some traumatic experiences might be more associated with complex posttraumatic reactions. For example, in our study experiencing physical abuse in childhood or poly-traumatization were associated with the risk for CPTSD, in comparison to PTSD or no PTSD/CPTSD risk groups. So it is necessary to keep in mind that people with the history of such experiences might be at greater risk of developing not only PTSD but also disturbances in self-organization symptoms. However, the results in different studies with various samples are not consistent, probably confirming the role of personal and environmental factors resulting in vulnerability or resilience to horrific life experiences (Maercker et al., 2022). This means that evaluating the reactions to potentially traumatic experiences might be more important than the type of trauma itself.

### Limitations and future directions

The findings from the present study should be interpreted in light of some limitations. The sample was relatively small, especially for the PTSD subgroup, and predominantly female. Furthermore, The Life Events Checklist-Revised (Weathers et al., 2013) used in this study measures exposure to specific traumatic events and type of exposure (e.g., witnessing, learning about the event). However, it has limitations in measuring various important aspects of traumatic experiences, for example, the repetitiveness of the event, the age of the participant during the exposure, etc. In the ICD-11, complex posttraumatic stress disorder is linked with prolonged or repetitive traumatic events. So analyses that include these additional factors of traumatic exposure might help to identify the role of specific aspects of traumatic experiences in predicting different posttraumatic disorders.

## Conclusion

The exploration of PTSD and CPTSD risk factors is essential for better recognition of these disorders. Since trauma exposure is a crucial component for diagnosing PTSD and CPTSD, it is important to determine if different types of traumatic events might help distinguish PTSD and CPTSD. The current study showed that both PTSD and CPTSD were linked with exposure to traumas of interpersonal types, such as sexual abuse in childhood or adulthood. Also, CPTSD was found to be related to childhood physical abuse and a greater number of experienced different traumatic events. We also found that traumatic events of non-interpersonal nature, namely experiences of natural disasters and serious accidents, were more related to PTSD but not CPTSD.

## Data availability statement

The raw data supporting the conclusions of this article will be made available by the authors, without undue reservation.

## Ethics statement

The studies involving human participants were reviewed and approved by Vilnius University Research Ethics Committee (16.01.2020, no. 33). The patients/participants provided their written informed consent to participate in this study.

## Author contributions

OG and EK designed the study. OG, MK, and AK recruited the participants and collected the data. AK conducted data analyses and prepared the first draft of the manuscript. MK, OG, and EK reviewed the manuscript. All authors contributed to the article and approved the submitted version.

## Funding

This study has received funding from the European Social Fund (project no 09.3.3-LMT-K-712-19-0048) under a grant agreement with the Research Council of Lithuania (LMTLT).

## Conflict of interest

The authors declare that the research was conducted in the absence of any commercial or financial relationships that could be construed as a potential conflict of interest.

## Publisher’s note

All claims expressed in this article are solely those of the authors and do not necessarily represent those of their affiliated organizations, or those of the publisher, the editors and the reviewers. Any product that may be evaluated in this article, or claim that may be made by its manufacturer, is not guaranteed or endorsed by the publisher.
